# Gutta Percha Splints

**Published:** 1854-11

**Authors:** Samuel H. Case

**Affiliations:** Oneonta, N. Y.


### GUTTA PERCHA SPLINTS.

BY SAML. H. CASE, M. D. ONEONTA, N. Y.

For many fractures, sprains, diseased and wounded joints, where something like an immovable apparatus is required, gutta percha as a material for splints is, above all other appliances and inventions with which I am acquainted, the very thing desired. Although it is several years since it was introduced into this country and recommended to the profession for various surgical purposes, I am not aware that it has come into very general use as an article for splints; indeed the probability is that it is not very frequently used.

These splints have advantages, however, over starch, felt, or paste board, in ease and facility of adaptation to the particular case in hand. They are sufficiently firm, and are not liable to be injured at all by water or any other application that the Surgeon might wish to make to the limb. They may be easily removed and re-applied whenever desirable, and altered in shape, if necessary, with very little trouble. The material is cheap and durable; it can be remoulded whenever wanted, thus superseding the necessity of keeping on hand a score of felt and curved splints of different shapes and sizes.

It may be a convenience to some practitioners if I describe here the manner in which I have been accustomed to mould these splints for fractures of the leg. Any other desired shape can be made in a similar way:

Put as much gutta percha as may be required in water, brought nearly or quite to the boiling point, and when soft enough to work, make a roll of it on a table; then bend a due proportion of the roll at a right angle for the foot, and flatten the whole with a rolling pin to the requisite length, width and thickness. It should be as long as the leg and foot, wide enough to envelop about half the circumference or even more of the limb, and from an eighth to one fourth of an inch in thickness. After making this "tablet" in proper shape, select some individual having a limb the size and shape as near as may be, of that of the patient—cover it with a stocking and perhaps cloths or bandages so as to resemble the fractured leg when bandaged, place the foot at a proper angle with the leg and request the individual to keep it so, till the splint shall be removed, then apply the "tablet in a yielding state (not too hot or soft, for it may stick to the stocking or bandages) and over all apply a roller bandage from the toes to the knee, so as to adjust every part of the splint to the limb. In fifteen or twenty minutes it will be hard enough to retain its shape and may be removed.

The fractured limb should be bandaged and the splint lined with cotton flannel or two or three folds of soft cotton or linen cloth before it is applied, otherwise the confined perspiration may irritate and excoriate the skin, the gutta percha being impervious to water. If this be not sufficient, holes may be punched or bored in the splint. For this reason too it is better not to enclose the limb entirely with these splints—a stout narrow one may be formed for the opposite side if deemed necessary, or a common padded splint may be used, over which the outside tapes or strips of bandage may be tied, and the whole secured with a sufficient degree of firmness.

